# Analectic

**Published:** 1830

**Authors:** 


### MISCELLANEOUS INTELLIGENCE.

### *ANALECTIC, ANALYTICAL, AND ORIGINAL.*

### I. ANALECTIC.

1. *Nerves of the Placenta.*—We find in the *Journal des Progres*, a paper on the nerves of the placenta, accompanied with a beautiful lithographic drawing, exhibiting the results of examtions made by Mr. BAUER under SIR EVERARD HOME's directions.

Sir EVERARD's researches on the incubated egg, led him to suppose the arteries are developed under the influence of nerves, and he accordingly engaged Mr. BAUER to investigate the subject, with the aid of those wonderful lenses which have given such eclat both to him and his patron.

Mr. BAUER was furnished with the placenta of a seal preserved in spirits, and as the vessels of the cord are not twisted in that animal, he would have a better opportunity of ascertaining the existence of nerves if they should really exist. They would have lost, also, their transparency by the coagulating power of the spirit, and consequently be less apt to escape observation. That gentleman did discover nerves accompanying the vessels of the cord, and also found and exhibited them in the uterine portion of the placenta.

Sir EVERARD HOME thinks that the discovery of nerves in the placenta leads fairly to the inference, that the brain of the fœtus has a real nervous connexion with that of the mother, may serve to explain why children have been born, affected with small pox and with intermittent fever ; and that we should be no longer astonished at the birth of acephalous fœtuses, since the nerves of the mother have had an intercourse with the organs of the monster. The influence of moral causes affecting the mother, and extending to her offspring, need no longer be considered as wholly unintelligible.

2. *Urachus.*—Dr. HOFMEISTER, in the Zeitschr, fur Nat und Heilk. Vol V. No. 6. relates the history of a singular case. A boy two years old had at the naval a fleshy excrescence which was fungous, red and pedunculated, through which the urine escaped whenever his body was bent backwards. The canal of the urethra was quite pervious, and admitted of the passage of urine. The pedicle of the fungus was secured with a ligature and fell off; the part was cicatrized, and the urine ceased to flow through the channel. It was thought to be the remains of the urachus.

3. *State of the Uteras eight years after the performance of the Caesarian Operation.*—The operation had been performed eight years before by Dr. Walther; the uterus was of its natural form and dimensions. At the external surface of the anterior parietes, a furrow three lines in length, indicates the place where the incision was made; the peritoneum is very firmly adherent to it.

The edges of the wound were contracted and turned in. The inner cicatrix was lower down, and half an inch longer than the outer one; it extended as low as the neck. The anterior parietes of the uterus in the neighborhood of the cicatrix was three lines thick; the posterior parietes opposite to it was four lines. The cavity of the uterus was perfectly natural, except that there was a very thin fleshy polypus at the neck. The left tube and ovary were perfectly natural; those on the right adhered to each other by plastic lymph.

The account of the uterus above described is given in Graafe and Walther's Journal by Mr. Mayer of Bonn.

4. *Lymphatics and Veins communicate with each other.*—At the Royal Institute of France, on the 17th of August, M. Dubled announced by letter, that he had filled the lower two-thirds of the thoracic duct and several lymphatic vessels by injecting the lower cava. He found it necessary to produce a considerable distension of the cava in order to obtain this result. He also made the following experiment. He tied the vena cava just below the diaphragm in a living animal; several hours afterwards he found blood in the thoracic duct and in some lymphatic vessels.

5. *Abnormal Menstruation.*—We obtain from the *Nouvelle Bibliotheque Medicale*, the following account of abnormal menstruation, which was published in Hufeland's Journal.

A woman ceased to menstruate at forty-one years of age, and became perfectly regular again at seventy-four. A woman aged sixty-one was still regular in menstruating, although she had begun at the age of thirteen years. She had not conceived for more than twenty years. She applied for medical advice on account of loss of strength. She took every day, for three months, two grains of alum, three of nitrate of potassa, and one-tenth of a grain of opium, sometimes with sugar and canella, sometimes in solution : the catamenia gradually diminished without occasioning any inconvenience in regard to her health. A Jewish child, eight years old, had discharged for three days about three desert spoonfuls of blood. One drop of Haller's acid elixir, half an ounce of distilled water, and the same quantity of syrup of canella, taken in doses of a desert spoonful morning and evening, occasioned this premature menstruation to cease without affecting the child's health.

6. *Wedemeyer on the Circulation.*—In the Quarterly Summary of our last number, we noticed Dr. WEDEMEYER's opinion, that the movement of the blood in the capillaries results chiefly from the action of the heart, and not of the great vessels. We shall now give his views on the question, whether the movement of the blood in them does at all depend on the contractility of the small arteries and of the capillaries themselves.

His researches led him to conclude with confidence, that the small arteries are endowed with a certain degree of contractile power. BICHAT had asserted that the mildest and most inert fluids could not, during life, be injected from arteries into veins in consequence of the contraction of the capillary vessels ; but MARGENDIE states that even irritating liquors can be forced through. Dr. WEDEMEYER has proved by his own experiments that water can be forced from arteries into veins during the life of the subject, but that the passage of very active irritants is retarded, and even totally prevented, by the contraction of the capillary intermediate vessels. He laid bare the brachial artery and humeral vein of a horse, and having cut them, pushed an injection of tepid water into the lower portion of the artery, and at every thrust of the piston, the water was seen to flow out of the lower portion of the divided veins, and to stop flowing when he ceased to inject into the artery : a very slight force only was required. On substituting cold alcohol for the tepid water, a much greater degree of force was required to make the fluid which escaped from the veins give evidence, by smell and taste, that it was the same as was thrown in by the syringe ; and in spite of the force, it did not escape in jets, but only oozed out. In another horse the experiment was tried with vinegar, and succeeded at first as well as when made with water, but the capillaries gradually contracted, so as finally to prevent the transmission of the acid entirely. These experiments were often repeated, and with the same or very analogous results, and led to the conclusion, that the capillary vessels are endowed with the power of self contraction in a very high degree, when violently stimulated ; a contractile power which is purely vital. If it be objected that the alcohol and vinegar were arrested by a chemical change induced by them, it is sufficient to observe, that after death they pass as readily as warm water.

Dr. Wedemeyer made many microscopic observations on the capillaries, as affected by the direct application of various stimulants. These are not exhibited in the *Archives*, which presents us, however, with a view of his experiments on the capillaries, made in order to ascertain the effects produced upon them by galvanism.

When small arteries are touched with either the positive or negative wire of a pile composed of from fourteen to twenty four pairs, we observe in from ten to thirty seconds, and sometimes at the instant of contact, a contraction of their parietes, which may amount to one-fourth, one-half, or even three-fourths of their diameter. The blood flows more rapidly in vessels thus contracted, but nearer to the heart the arteries are dilated, and the course of the blood less rapid. Sometimes this contraction lasts for hours together, but at others for only about ten minutes, after which the vessels recover their caliber. A second application most commonly produces no apparent effect. He never observed that the small veins, subjected to this experiment, were contracted, except in one solitary instance ; while, on the contrary, in every other case, they were gradually dilated, and the flow of the blood in them became slower and slower, or even stopped completely. The effect (secondary,) of galvanism on the capillaries was still more remarkable, for in the course of a minute the flow of the blood became very much diminished, was wholly suspended, and that fluid was actually coagulated : the capillaries appeared to be dilated, and those which were before colourless and transparent, were soon found to be of a lively red, as if inflamed.

He regards the contractility proved by the experiments to exist, as analogous to that in the urethra, in the small ramifications of the bronchiæ, in the corion of the skin, &c. It is never manifest in the ordinary state of the circulation, but only on the application of certain stimulants. It does not contribute at all to the movement of the blood in the capillary vessels, but, on the contrary, as Haller remarked, serves rather to retard and embarrass it. The same he thinks to be true of the small arteries. He says that the motion of the blood in the capillaries is arrested as soon as we tie up the aorta near the heart, and that those who have made a statement contrary to this, have been misled by particular circumstances.

7. *Case in which no Division existed between the Ventricles of the Heart.*—A case of this kind is related by Dr. Wittchke, in Hufeland and Osann's Journal for April, 1828.

The patient was twenty-four years of age; had from his infancy been affected with a peculiar palpitation of the heart, unattended by any other symptom of disease. Five years ago, after an inflammation of the chest, the palpitation augmented in violence; he appeared sometimes to be in danger of suffocation. The palpitation and sense of suffocation abated when he sat upright, and pressed his breast firmly against any hard body. Medical treatment rendered him no relief; the occurrence of hemorrhoids appeared, however, in some measure to render his sufferings more tolerable. Finally, the accessions of suffocation became more violent, particularly at night; the only position in which the patient could find any ease was sitting upright. By degrees œdema of the feet showed itself, and extended to the legs; at length the abdomen became distended from effusion, and the scrotum was swollen to a great extent. His countenance was livid, swollen, and of a doughy appearance. The upper extremities presented the same phenomena.

He was first visited by Dr. W. on the 17th of November, 1825; he found him lying in bed, with his head and shoulders greatly elevated, and unable to rise. The preceding night he had suffered a very severe attack of suffocation; for fear of a return of which, he begged, with tears in his eyes, that the doctor, by puncturing the abdomen, would relieve him of the load of water which it contained, and thus enable him to assume the erect posture. His carotids throbbed violently; the heart beat with such force that the whole of the left side of the chest was shook by its action. The motions of the chest were distinct, commencing above and extending downwards. The pulse was regular, somewhat tense, and moderately full, ninety in a minute. The respiration was rattling, as though some obstruction existed in the trachea. The patient could speak only with great difficulty; the tone of the voice was hoarse and low. The body presented all the symptoms of extensive general dropsy. Appetite and digestion not particularly affected, the tongue very red and clean; great thirst; the skin had the feel of dry parchment. The skin on the fore part of the scrotum, being irritated by the passage of the urine over it, presented an erysipelatous appearance; as also did the calves of the legs. The patient complained of considerable pain of the left thigh.

Although the dropsy was viewed as a mere symptom yet with a view to the mitigation of his sufferings the abdomen was punctured. Diuretics and other palliative treatment were prescribed with temporary relief, but the patient died on the 30th.

8. *Sectio cadaveris.* The thorax only examined; it contained about a pint of bloody serum. Lungs healthy, but pressed backwards, in consequence of the augmented size of the heart; they were not greatly distended with blood, and nowhere adhered to the pleura. The pericardium adhered completely to the surface of the heart, with the exception of that part which is next the diaphragm, where not a trace of it was discoverable, the heart lying immediately upon the latter. The heart was at least three times its normal size, and filled with a considerable quantity of black half coagulated blood; the parietes of both ventricles being of a thickness proportionate to the augmented bulk of the organs. The most remarkable circumstance was the absence of the septum ventriculorum, of which not a trace remained; notwithstanding which, the openings of the great vessels were entirely natural, excepting that those of the veins were somewhat enlarged; the valves perfect. The auricles, with the exception of their increased size, were also in every respect natural. The aorta arose very straight, and was filled with a fibrous coagulum. The veins were collapsed, and contained a small quantity of fluid blood; this was also the state of all the great veins of the chest. The ductus arteriosus was, as usual, entirely closed.—*North American Med. and Surg. Journal.*

9. *Influence of the Male extending beyond a single Impregnation.*—A seven eighths Arabian mare, belonging to the earl of Morton, which had never been bred from before, had a mule by a quagga: subsequently she had three foals by a black Arabian horse. The two first of these are thus described: "They have the character of the Arabian breed as decidedly as can be expected where fifteen sixteenths of the blood are Arabian, and they are fine specimens of that breed; but, both in their color and in the hair of their manes, they have a striking resemblance to the quagga. Their color is bay, marked more or less like the quagga in a darker tint. Both are distinguished by the dark line along the ridge of the back, the dark stripes across the forehead, and the dark bars across the back part of the legs. Both their manes are black; that of the filly is short, stiff, and stands upright; that of the colt is long, but so stiff as to arch upwards, and to hang clear of the sides of the neck; in which circumstance it resembles that of the hybrid. This is the more remarkable, as the manes of the Arabian breed hang lank, and closer to the neck than those of most others."* Mr. Mayo, who quotes this curious fact in his excellent "Outlines of Philosophy," observes, that "a similar occurrence to the preceding

*Philos. Trans. 1821, P. 21.

is mentioned by Mr Giles, respecting a litter of pigs, which resembled in color a former litter by a wild boar. The best explanation of these phenomena is to suppose that connexion with the male produces a physical impression, not merely upon the ova which are ripe for impregnation, but upon others, likewise, that are at the time immature. In gallinaceous birds, in turkeys, for instance, it is well known that a single coitus will actually impregnate all the ova that are laid during the breeding season. The explanation which I have offered seems to me far more reasonable than any supposed influence of the imagination, the effect of which on any occasion, even in human beings, appears more than doubtful."—*Mayo's Physiology, 2d ed.* p. 489.

10. *Congenital Absence of the Iris without loss of Vision.*—The congenital absence of the iris, without loss of the sense of sight, is one of the most uncommon and singular organic anomalies. M. Rudolphi, (Grundriss der Physiologie, t. ii. part i. p. 221,) has doubted whether it has ever been observed; but we are now in possession of many facts which leave no room for incredulity. The change from the natural state to the total absence of the iris appears to be formed by congenital irregularities in the figure of the pupil. The first degree of anomaly is constituted by the oblong and perpendicular pupil, like that of the cat. Another preternatural formation consists in the division of the iris from the inferior margin of the pupil to the union of the cornea with the sclerotic coat.

Dr. Behr briefly reports all the known cases of total absence of the iris. Klinkosch, (Programma quo sect. et demonsta. indicit. Prague, 1766; Meckel, Pathol. Anat. t. i. p. 395,) first observed this anomaly, but in a case where there also existed organic malformation of the eye and the whole body. The first case of complete congenital absence of the iris, without any other complication, was communicated to the Societe du Cercle Medical de Paris, by Mr. A. Morisson, of London, (Nouveau Journ. de Med. &c. t. vi. p. 105; M. Baralta has described two eyes, in each of which the iris was wanting, (Praktische Beobacht. uber die vorzuglichsten Augenkrankheiten.) Professor Dzondi mentions a similar case, (Rust, Mag. f. d. ges. Heilkunde, t. vi. p. 33; and another is reported by Dr. Pænitz, of Dresden, (Zeitschrift fur Nat. und Heilk. t. ii. p. 214.) Lastly, M. Behr gives us the details of the following case, which he himself saw.

Caroline Schwabe, born in 1826, from the first day of her life, was so sensible to the impression of light, that she cried loudly whenever any luminous rays fell upon her eye. Her mother could perceive nothing peculiar in her eyes; but M.

Behr, upon examining them in May 1827, discovered a total absence of the irides. The eyes of the mother and father were blue, and naturally formed. The child presented no other irregular formation, excepting that the upper eyelids were thick and swollen, and the eyebrows covered with light weak hair. By degrees the infant became accustomed to light, but the eyes were always very mobile, and agitated in their orbits. The cornea was rather more convex than usual. In November, 1827, she was attacked with measles, accompanied by excessive sensibility of the eyes. September 1828, the child could direct the eyes steadfastly to any object, and the sclerotic was now seen of a bluish tint, and the large pupil of a deep black colour. When the child was placed at the extremity of a room, and rays of light were directed through the window upon the eyes, a phosphorescent redness was perceived, which gave to the eye the aspect of a luminous ruby, or of a burning coal. The visual faculty did not appear to be affected by this anomalous structure of the eyes. The child, however, seemed to be more comfortable in the weak light of the evening: she was then more cheerful and playful than at other times. She could see also in almost total darkness. The brightest colours, as red or yellow, were the most agreeable to her. If she wished to examine any minute object, she drew it very near her, and always placed it below the visual axis. It appeared painful to her to look upwards, even in a weak light. The other senses were perfect, and her hearing was remarkably acute.—*Lond. Med. and Phys. Journ. Jan.* 1830, *from Hecker's Ann. April*, 1829.

11. *On the Regeneration of Bone.*—M. Flourens read a communication to the Royal Academy of Sciences, at their meeting on the 20th July last, on the regeneration of bone. M. F. has made a number of experiments on young birds, principally pullets, and the following are the results at which he has arrived. 1st. If the external periosteum of a bone of the cranium be removed, the external lamina of this bone becomes necrossed, and is thrown off; in this case a new periosteum is first formed, afterwards a cartilage, which subsequently ossifies. 2d. If a bone of the cranium and its periosteum is removed entire, the dura mater remaining perfect, this latter membrane reproduces only the internal lamina of the bone; the external is reproduced, as in the preceding case, by a periosteum newly formed. 3d. If the periosteum, the bone, and dura mater are removed, a new periosteum and a new dura mater are first formed, afterwards a double cartilage is produced between these two membranes, which is finally converted into two osseous lamina. 4th. All bones are not susceptible of reproduction; those which the author has seen reproduced in his experiments are, the frontal, the occipital, the parietal, and the other bones of the vault of the cranium; but the osseous envelope of the semicircular canals, and these last themselves are not reproduced. Nevertheless if a canal has been only divided, the two ends reunite after a time and are connected by a bone which at that point obliterates the cavity of the canal. M. F. gives the following account of the mechanism of this reproduction. 1st. It is always the periosteum or the dura mater which is first reproduced, and they afterwards reproduce the cartilage and the bone. 2d. It is always the old periosteum and the old dura mater, which give birth to the new periosteum and new dura mater; it is also at the borders that the new organization always commences. 3d. The new bone is never as regular in its structure as the primitive one; the two lamina of which it is composed are often confounded together, or at least are only separated by an imperfect diploe. 4th. An effusion of organizable lymph, at the border of the part which forms it, always precedes a new progress of formation.—*Archives Generales, Oct.* 1829.

12. *Instance in which Life was supported for a length of time by the Absorption of the Fluids of the Body.*—A very curious instance of life being supported by the absorption of the fluids of the body, is related by Mr. Granger, in his *Elements of General Anotomy*, as having occurred some years since at Dover. "A hog weighing one hundred and sixty pounds, was buried under a portion of the cliff, which fell on its stye, for the long space of one hundred and sixty days. At the end of this time, being dug out, it weighed only forty pounds, and was extremely emaciated, clean, and white. As there was neither food nor water in the stye, when the cliff fell, this hog must have existed during the time mentioned, by the removal of the adipose and other fluids from their containing structures into the circulating system."—*American Journal.*

13. *Loss of Sensation in one-half of the Body without Loss of the Power of Motion.*—Notwithstanding the numerous researches of modern physiologists, and the ingenious experiments they have instituted to elucidate the mechanism of the functions of the nervous system, we are still ignorant what portions of the brain are the exclusive seat of sensibility, and what determine the movements, though observations similar to the one we are about to notice, show that these faculties are entirely isolated. The following interesting case, related by Dr. Le Sauvage, of Caen, in the *Archives Generales*, for November last, affords a very remarkable example of the possibility of the loss of sensation without the locomotive faculties suffering in the slightest degree. A man aged seventy-three, well made, habitually enjoying good health, had perceived for fifteen days some giddiness, when on the 10th of March, 1828, being about fifty steps from his house, he experienced suddenly a numbness in the whole of the left lower extremity. It appeared to him that his foot sunk deeply, and he seized his thigh with both hands, as if to prevent its sinking into the ground. Almost at the same instant, the numbness extended over the whole left side. Alarmed at this unusual condition, he became anxious to get home, and ran with facility that distance. The next day Dr. Le Sauvage was sent for, who found the patient in the following condition; the intellectual faculties natural; the pulse nearly the same in both sides of the body; he could walk, move his arms, and take hold of objects without difficulty, only the elevation to which the arm could be raised by the deltoid was a little limited; but he had no consciousness of the movements he performed, nor of the bodies he touched. The skin over the whole left side was absolutely insensible; he might be pinched and pricked on this side ever so much, without his perceiving any pain, or even without his being aware of it. At the anterior part of the body, the median line was not the precise limit of the sensible parts. On the left side, the skin possessed sensation to the extent of about an inch from this line: beyond this point there was complete insensibility on this side. On the affected side, the sight and hearing were perfectly natural, but the senses of smell and taste were lost. When the left half of the tongue was wet with strong vinegar, the patient did not taste it in the least, whilst the taste of this fluid was strongly perceived by the other half of this organ. Strong odours placed under the left nostril were not perceived, except when the patient made a strong inspiration, but then the odour passed to the right nostril through the posterior nares. When the hand was placed upon the head, the patient felt only that portion resting on the right half, &c. This affection has hitherto resisted all treatment. The constitution of the patient, however, does not appear to have suffered any injury.

14. *Intermittent Aphonia.*—A very remarkable case of this is related by M. Rennes, Professor in the Military Hospital of Instruction at Strasbourg, in the *Archives Generales*, for June last. The subject of the case was a Madame M. a brunette, of middle size, aged forty-six, married at the age of twenty, residing constantly at her country seat, at Pengord, in the midst of a wood, where the air is good, presenting nothing in her physical constitution, or in her character, which is extremely cold, that could lead to a suspicion of a predominance of her nervous system. Her regimen has always been regular; her life tranquil; she never had any violent disease; she has had two children, the youngest is twenty-one years of age. This lady was attacked annually, from the age of thirty-three years, with aphonia. These attacks were abrupt, and came on at noon, unaccompanied at first with any local or general uneasiness. The loss of voice was sudden and complete, and lasted till midnight of the same day, when speech returned in the same rapid manner in which it was lost. This occurred every day for about three weeks, and then ceased. Three of these accessions, each of a fortnight or three weeks duration, took place in the course of the first year, 1812, and the same course obtained for a few years afterwards. In 1819, the periodicity became more regular. Each year, in the month of February, the approach of the malady was announced in the morning, by wandering pains, shiverings, and horripilations. Exactly at midday the voice became extinct, without any affection of the pulse, any sense of constriction about the throat, but only a sense of tightness and uneasiness in the epigastric region. She would go to bed at nine o'clock, and awake quite well, to go through the same process the succeeding day. Sometimes, however, there were accompanying febrile symptoms, head-ache, &c. but these were rare. She had no perspiration. Nevertheless these attacks diminished her strength, and caused considerable loss of flesh. She had been bled—had taken sulphate of quinine, and various other medicines, without the least effect. Of late years the duration of these attacks has varied from three to seven months every year. The attack over, she regains her embonpoint and strength, and appears as if she had never been unwell.—*American Journal.*

15. *Intermittent Aphonia of thirty years standing.*—This is related by Dr. Ollivier, of Angers, in the same journal with the preceding case. Maria Louisa Girou, midwife at the Hotel Dieu of Angers, aged forty-four, (at the period when she came under observation, 1818,) of a nervous and sanguineous temperament, delicate constitution, had menstruated regularly from the age of fifteen to that of eighteen, when she had profuse menorrhagia, occasioned by some violent mental emotions. About this period she became affected for the first time, and without any evident cause, with complete aphonia, which lasted several days. From this period the complaint came on at irregular intervals—sometimes several times in the month —sometimes with intervals of many months. She had once an immunity of a whole year. There was no apparent disturbance of the constitution at the periods of attack. They commenced with a sense of irritation in the throat, which gradually increased till the voice was totally lost, when the irritation always subsided. During the period of the attack, the patient felt a sense of oppression about the region of the heart, and of heat ascending in currents to the head. In all other respects she was well, and went about her avocations as usual.

From the age of eighteen to that of forty-four years, the attacks of aphonia have always presented the same phenomena; but latterly, since menstruation has ceased, the intervals between the accessions are longer. Various remedies, chiefly of the antispasmodic and tonic kind, had been employed, without making the slightest impression on the malady. At length blood-letting was tried—and scarcely had an ounce flowed from a vein when restoration of the voice took place. This remedy has invariably produced the same effect in every subsequent attack of aphonia. Leeches gave the same relief, by the time that an ounce or two of blood was abstracted. M. Ollivier himself was witness many times of this extraordinary efficacy of bleeding. The patient felt as if a load were removed from her heart whenever the blood flowed, and then her voice was restored.

16. *Paralysis of the Muscles of one side of the Face from disease of the Portio Dura.*—Mr. Abercrombie, in his work on the brain, makes the following very interesting observations on this affection; they merit the attention of the profession. We have seen very many cases of this complaint, the pathology of which is not generally understood, and have seen one case at least in which it was rendered permanent by mal-treatment. "The important practical application of the discoveries of Mr. Bell," says Mr. Abercrombie, "is, that there may be paralysis of the muscles of one side of the face, producing distortion of the mouth with inability to shut the eye-lids, without disease of the brain, and consequently without danger. This affection depends upon a disease limited to the portio dura of the seventh nerve, and may be produced by inflammation of the ear or the parotid gland, or tumours compressing the nerve on any part of its course. The most common example of it seems to originate in a kind of rheumatic inflammation produced by cold, especially by exposure to a current of cold air, as when a person has sat long, or has slept opposite to an open window, or has sat in a carriage with a cold wind blowing on one side of his head. It is to be treated chiefly by local remedies, as topical bleeding, blistering, and the application of warm water or steam. In this manner it is often speedily removed, but in some cases proves tedious, and does not go off entirely for several months. The affection is of course still more untractable, or even permanent, when it depends upon a permanent cause, such as tumours compressing the nerve, or destruction of a portion of the nerve by wounds or extensive suppurations. There is also a very formidable modification of it which depends upon disease of the temporal bone.

"The character by which these cases are distinguished from paralysis depending upon disease of the brain, consists chiefly in the sensibility of the parts remaining unimpaired. The loss of motion also is confined to the muscles of the face and eyelids, and does not affect those of the jaw. These peculiarities arise from the remarkable facts discovered by Mr. Bell, Mr. Shaw, Mr. Mayo, and others, that the portio dura of the seventh is a nerve of motion only, supplying the muscles of the face and the orbicularis of the eye, but not the muscles of the jaw; and that the sensibility of all these parts, and the motion of the muscles of the jaw are derived from the fifth, which, having a double origin, is a nerve both of sensation and motion. An important distinction, however, is to be kept in mind in regard to the paralysis of the eye-lids which occurs in these cases, namely, that it is the inability to shut the eye that arises from the affection of the portio dura of the seventh. The dropping of the upper eye-lid and inability to raise it, is a disease entirely of a different nature; it depends upon an affection of the third nerve, and consequently gives more reason to suspect disease within the head.

"When therefore we find paralysis and distortion of the face, with loss of sensation of the parts, we have reason to suspect disease within the head, the portio dura of the seventh and the fifth being both affected. But when we have the paralysis without diminution of sensation, the disease depends upon an affection of the portio dura alone, and may be entirely without danger. Such cases however are not to be treated lightly, but the cause of them ought to be carefully investigated; for if there be any reason to suspect that the affection depends upon disease of the temporal bone, it may come to be attended with danger by inflammatory action spreading inwards to the dura mater or brain. There is another modification also which requires to be watched with anxiety, namely, when the affection is accompanied with deafness; as this gives reason to believe that both portions of the seventh nerve are affected, and consequently to suspect an internal cause."

17. *Dupuytren's Method of Removing Fibrous Polypi of the Uterus.*—M. Dupuytren, instead of the method hitherto employed for the removal of uterine polypi, extirpates them in all cases with the knife. He has been led to prefer this operation, from the consideration of the fibrous nature of polypi, the facility with which by moderate but continued traction the uterus may be drawn even with the vulva, the fact that hæmorrhage is less to be feared than is supposed, and that the difficulties arising from the deep situation of the pedicle of the tumor may be easily overcome. Besides, it is sometimes impossible to apply a ligature, either because the tumour is too deeply situated or completely fills the vagina, and even greatly distends it, so that the fingers or instrument for applying the ligature cannot be introduced; moreover the consequences of the application of the ligature are often very dangerous. The following is M. Dupuytren's mode of operating. The patient is placed on her back upon the edge of a bed, her legs and thighs flexed and fixed as for the operation of lithotomy. Very strong dressing forceps are introduced into the vagina, with which the polypus is seized hold of, if it do not project beyond the vulva; the forceps are introduced on the finger or with the aid of a speculum; in this last case their branches must be straight and the rings small, so that they can pass into the speculum, when the latter is withdrawn from the vagina, after the polypus has been seized hold of by the forceps. The surgeon then employs gradual traction to draw out the polypi s, or if it already project beyond the vagina, to expose its pedicle. Sometimes a single forceps is not sufficient, the first is then confided to an assistant, who continues the tractions, and the surgeon takes hold of another part of the polypus with another pair of forceps, and thus the desired effect is produced. The pedicle being brought to the orifice of the vagina, the surgeon ascertains the size of the pedicle and whether there be in it any large arteries, the latter is ascertained by the pulsation. If there be pulsations, a ligature is to be applied above the place where it is intended to make the incision, but where no pulsations are discovered, the pedicle is to be divided with a bistoury or scissors curved on both sides. When there is no pedicle, M. Dupuytren makes around the anterior half of the base of the tumour an incision which penetrates into the mucous tissue, the sub-mucous cellular tissue, or even the proper tissue of the uterus; a similar incision is made posteriorly, the two incisions united, and the tumour dissected out. In this case the operation is more complicated, and may be more readily followed by hemorrhage, inflammation, &c. M. Paillard, who reports this method in the *Revue Medicale* for June, 1829, states that numerous successful cases attest the advantages of this operation. It may be conceived that when these tumours are soft or have degenerated so as to tear easily, that it will present obstacles to the operation, but with patience, prudence, and slow and moderate traction, M. P. says that the surgeon will eventually succeed. Experience proves, he says, that dangerous hemorrhage never occurs; once only M. D. has been obliged to have recourse to the tampon, which readily arrested the hemorrhage.

18. *Extirpation of the Uterus.*—In our last, at p. 225, we gave some details of this operation as performed by M. Recamier, in July last, at the Hotel Dieu. Examination of the removed organ confirmed the diagnosis established by accurate investigation of the pelvis before the operation, and proved that all the diseased tissues were removed—a point essential to success. From the *Archives Generales* for October, we learn that M. Roux has since performed two similar operations, the details of which are given. In the *first*, the patient was 55 years of age, and had a cancer of the neck of the uterus, and after a careful examination by MM. Recamier, Dupuytren and Roux, the ablation of the uterus was regarded as the only chance of safety for the female. It was accordingly performed by M. Roux on the 20th of September last at 9, A. M., in the manner indicated in our last number. In this case, tumours on the anterior part of the neck of the uterus, rendered the separation from the bladder tedious and difficult ; and, after the ligatures were applied to the lateral ligaments, prevented the antiversion and descent of the organ, until part of the perineum was divided. Very little blood was lost. The operation lasted half an hour.

"The patient was much exhausted, pulse frequent and very feeble ; in half an hour there was some reaction, which was assisted by warm drinks and chicken water. In the evening the abdomen became painful ; six leeches were applied to the hypogastrium ; they were suffered to bleed but little, in consequence of the weakness of the pulse and the general pallor. The patient remained in a state of stupor, from which she could not be roused ; death occurred on the 21st at 6, P. M."

*Necropsy.*—The pelvis contained 2 oz. of serosity ; no evidence of peritonitis or any other inflammation. The left ovary was soft, and transformed into an encephaloid substance. On the right there were numerous encephaloid tumours more advanced. The posterior surface of the bladder presented a round opening, at least four lines in diameter.

In the *second* case, the patient, aged 38 years, was admitted into La Charite on the 25th of August last, with a severe cancerous disease of the uterus. After many examinations by experienced surgeons, M. Roux operated on the morning of the 25th of September.

In this case also, the antiversion of the uterus was executed with great difficulty, and gave rise to severe hemorrhage. The right ligature broke and was not replaced. The operation lasted near thirty minutes, and the patient suffered much.

Immediately after the operation, the patient was pale, cold; the pulse small, frequent and threadlike, and there was a slight scarlet coloured hemorrhage per vaginam. No reaction ensued; some tonics were administered, but ineffectually; and death occurred on the 26th, at 10, A. M.

*Post Mortem.* The pelvic cavity was found to contain a small quantity of blood; the serous membrane was of a rose colour, and the subjacent cellular tissue was already becoming friable. The bladder, rectum, and other viscera were sound. The left ligature was found embracing exactly the broad ligament at its inferior part. On the right side, the exact source of the hemorrage could not be discovered.

In the *Journ. Gen.* for October, the cases of Recamier and Roux are given with all the necessary details, and the learned editor, M. Gendrin, has compiled a history of all the cases in which the uterus has been extirpated, where there had been no prolapsus or retroversion of the organ. In Germany, although frequently proposed, and made the subject of special investigation, extirpation of the uterus in situ has been performed but once, and that in 1821, by M. Sauter, physician at Constance. The patient recovered from the effects of the operation, but died of a cancerous disease. In England it has been executed six times; four times by Mr. Blundell, once by Mr. Banner, of Liverpool, and once by Mr. Lizars. One of Mr. Blundell's succeeded; the result of Mr. Lizarr's is yet unknown.

In France it has been performed three times, as already detailed.

Of these ten operations, the result of one is unknown; three patients of the remaining nine recovered; and of these, one has since died of a return of the cancerous affection. In all there was immediate and great depression of the vital powers; and in no case has there been any hemorrhage sufficient to cause the fatal result. Those that died, have perished during the two first days, not from inflammation, but from prostration. In two cases, one of Sauter's and one of Roux's, the bladder was opened during the operation. M. Gendrin proposes a new method of operating, which he thinks would be superior to those given. But we have no room for further details.

This severe and dangerous operation of extirpating the uterus in situ, would seem at present hardly justifiable, except in cases not only favorable in themselves, where all the diseased structure can be removed, and the patient's health not too much impaired, but where the patient *solicits the attempt.* We doubt the *morality* of placing life in such immediate and imminent danger, even for the chance of ridding the patient of a disease fatal in its tendency, but which in most cases but slowly undermines the powers of the system. More force is imparted to this observation when we reflect on the uncertainty of the diagnosis as respects the extent, and even the nature of the disease, and the worse than useless attempt at extirpation, when adjacent tissues are also involved, as occurred in one of the above recorded cases. Fortunately, in America, the opportunity for such operations is rare, and we may safely and quietly wait the result of European experience, which promises to be extensive.—*N. A. Medical and Surgical Journal.*

19. *Croup in an Adult.*—So comparatively unfrequent is the croup in adults, that many have erroneously supposed it to be a disease confined entirely to children. At the session of the Royal Academy of Medicine, September 22, 1829, however, M. Tonnele, internal student of the Hospital for Children at Paris, read, in the name of M Desormeaux, a well marked case of croup in a married woman. It is stated that towards the close of her pregnancy, she was attacked with a pseudo-membranous angina. Delivery took place during the disease; the infant was perfectly healthy, and has so continued. The mother, however, died; and, on opening the body the membranous exudation peculiar to croup was found in the larynx and trachea; The lungs were in a state of engorgement, and the bronchiæ were the seat of intense inflammation.

20. *Wax as an application to Ulcers.*—The application of wax to old ulcerated legs has been practised in the Westminster Hospital, with great success, within the last few months. In every instance it has rapidly improved the character of the sore, and brought on a disposition to skin over, and in the greater number of cases the ulcers have healed in a much shorter time than could have been calculated with the ordinary applications—indeed it has succeeded often when all the usual dressings had been tried and failed.

21. *Tinea Capitis.*—There are perhaps few practitioners who have not been occasionally foiled in their attempts to cure this disease. The stimulating ointments and applications usually recommended, we have found very frequently to fail in affording relief, and not unfrequently they aggravate the mischief. We have been most successful with the physiological practice—treating the disease like other inflammations, with leeches, and then applying a poultice of flaxseed mucilage to the scalp, covering it with an oil-silk cap. After all inflammation has been subdued by these means, a moderately stimulating ointment may sometimes be found useful. In the last number of the *Edinburgh Medical and Surgical Journal*, Dr. M'Fadzen recommends a lotion of the acetate of lead, which Dr. Macartney also says will cure the eruption. The only thing to be feared, says Dr. M'F. is its being healed too suddenly. The following case, in which the remedy was successfully employed is given. A boy, aged eleven, of a flabby habit, and subject to a cough, has been labouring for some years under *Tinea Capitis*, or the *Porrigo scutulata* of Dr. Bateman, which has resisted all the common remedies, both local and constitutional, not only under my own care, but by the advice of others. The head having been shaved and well washed with soap and water, which exposed a number of inflamed irritable patches, covering about half of the hairy scalp, I dressed the head according to Professor Macartney's plan, namely, covering the diseased scalp with charpie dipped in a solution of the acetate of lead, (gr. iij. ad ozi. Aq.) and applying over all a well-fitted oiled silk cap, leaving directions to wash the lint three times a day in cold water, in order to free it from the secretions of the skin, reapplying it to the head, wetted with the medicated lotion. He has called upon me this day, (July 26th,) when I was happy to find him nearly cured, the inflamed and irritable patches having been replaced by a comparatively healthy skin. No constitutional treatment whatever was resorted to.

22. *Case of Fracture and Depression of the Inner Table of the Cranium by a musket ball, the External Table being uninjured.*—The following singular and interesting case, which occurred in the practice of Staff-surgeon Cooper, is related by Dr. Hennen, in his Military Surgery. The patient was struck at the battle of Waterloo, with a musket ball, on the right parietal bone, which was exposed, and had no appearance of being fractured; as, however, the symptoms of compression were urgent, and the patient was nearly in a lifeless state, Mr. Cooper conceived it right to apply the trephine to the part on which the violence had acted. He had not sawn long before the external table came away in the hollow of the trephine, leaving the inner table behind, which was not only splintered, but driven at one point more than half an inch into the membranes and substance of the brain. No sooner were the fragments taken out with a pair of forceps, than the man instantly sat up in his bed, looked around, and began to speak with the utmost rationality. It is a most extraordinary fact, that this patient got up and dressed himself the same day, without leave from the medical officers, and never had a bad symptom afterwards.

23. *Dislocation of the Clavicle forwards.—Reduction at the Expiration of Twelve Weeks.*—A man aged 50, fell with his arm outstretched in such a manner that it bore the whole brunt of the violence. Inability to raise the arm as before, and "an *intense dull swelling*, which after a time became almost imperceptible from the enormous degree of tumefaction which ensued," were the consequences of the accident. No attempt at reduction was made, and twelve weeks after the occurrence of the accident he entered the Winchester Hospital under the care of Mr. Lyford. On the superior part of the sternum was a distinct obtuse projection, exquisitely sensible when touched, and attended with slight inflammation of the integuments; this projection could readily be traced as a continuation and termination of the clavicle, though the motions of the shoulder appeared to produce no alteration in its situation. The motions in question were painful; the shoulder itself had a decided inclination forwards, and the distance between its point and the mesial line of the sternum, was shorter, on admeasurement, than in the opposite extremity.

"The treatment consisted in the application of the clavicle-bandage with pads under the axilla. The shoulders were drawn backwards, as far as they could be, which was not, however, to the fullest extent, the patient having acquired, from his agricultural pursuits, a somewhat prominent back, so that the bases of the scapulæ were farther asunder than natural. The effect of the bandage was not that of restoring the dislocated extremity of the clavicle immediately into its proper receptacle on the sternum. This object was accomplished, in the most gradual manner, by tightening the bandage every four or five days, until the scapulæ were completely approximated. The patient was confined in bed for three weeks on his back, which greatly assisted the bandage in its office of retaining the shoulders in the wished for position. Moderate pressure was made by the application of soap-plaster on the dislocated parts; and, at the expiration of a month, the parts had acquired their original and natural situations."—*Med. Chirug. Rev. Jan.* 1830, *from the Provincial Medical Gazette, No.* 11.

24. *Nicotin, the active principle of Tobacco.*—We derive the following statements from the *Archives Generales* for November last, which refers to *Geiger's Magazin fur Pharmacie* of December 1828. MM. Posselt and Reimann have described a new vegetable alkali in tobacco, for which they propose the name of *nicotin*, but which we should preferably designate by that of *nicotia.*

The process by which they obtained the new principle is sufficiently simple. They boiled twelve pounds of tobacco leaves in water acidulated with sulphuric acid, evaporated the filtered decoction by a gentle heat, and treated the residue with alcohol. The tincture was then distilled so as to separate the pure alcohol; and the aqueous residuum, after the addition of hydrate of lime was submitted to a new distillation. To the product thus obtained ether was added, the mixture was well shaken, and the ether then distilled off: another portion of ether was added, which was also distilled; and the process was repeated till the residuum ceased to present an acrid taste. The several portions of ether which came over were mixed, and treated with chloride of calcium: and the anhydrous ethereal solution thus procured was distilled by means of a salt water bath. The result of this operation was the nicotin. The authors procured two drachms of this alkaline principle from the 12 lbs. of tobacco which they employed.

Pure nicotin is liquid at about 21deg. of Fahrenheit. It is transparent, of a reddish brown colour, and of a pungent odour similar to that of dry tobacco. The smell is rendered more penetrating in proportion to the elevation of temperature. Its taste is extremely acrid and burning, and remains long upon the tongue. When dropped upon paper it produces a greasy stain, which, however, completely disappears in the space of two hours. It is volatilized upon exposure to the air, leaving a slight residue of resinous matter. It boils at 474deg. F., rises in white vapours when heated to near the boiling point of water, and takes fire upon the application of a lighted match. It is heavier than water.

Nicotin is soluble in water in all proportions, and its solution presents manifest signs of alkalinity.—*N. A. Med. & Sur. Jour.*

25. *Jalap the product of an Ipomœa.*—The question whether the true jalap plant is a convolvulus or ipomœa seems now to be settled. Professor Coxe, of the University of Pennsylvania, having procured from Xalapa a number of the plants in a growing state, placed them in his garden, and succeeded with great care in bringing them to such perfection, as to authorize a decision upon their botanical character. He has no hesitation in pronouncing the plant to be an ipomœa, and is supported in his decision by the opinion of Mr. Nuttall, one of the first botanists of this country. A paper by Dr. Coxe, upon the subject, is contained in the American Journal of the Medical Sciences for February last.

26. *Strychnia in Paralysis.*—It appears from the experiments of Desportes, Magendie, Delile, Orfila, and others, that the nux vomica does not occasion any organic lesion in the animal frame, though it has a direct action upon the nervous system, causing death from asphyxia produced by the immobility of the chest during the violence of the tetanic spasms of the thoracic and abdominal muscles. Frouquier was led to make trial of the strychnia in several cases of paralysis of the lower extremities, and published his successful results in that disease; and many others have employed the remedy with various, if not doubtful issues. Dr. Bardsley details 23 cases of paralysis treated with strychnine, and gives a tabular view of twelve more. We shall glance at some of these in a condensed form.

*Case* 1. A female aged 30, had entirely lost the power of the left side, with diminished sensibility, occasional head-ache and vertigo—articulation impaired—urine and fæces passed involuntarily—face drawn a little to one side—pulse feeble at 86—countenance pallid. The attack had occurred three months previously, shortly after parturition, and had gradually increased. Attributed the complaint to cold and over fatigue when far advanced in pregnancy. Leeches, blisters, and aperients having been premised, the strychnine was exhibited in doses of the twelfth part of a grain twice a day, and afterwards increased in frequency and quantity. It was more than three weeks before she began to feel the influence of the medicine in slight pricklings of the paralyzed limbs. The sensation began to revive—smart convulsive twichings occurred—motion improved—she had some command over the bladder and rectum—and finally recovered completely. The strychine amounted to a grain twice a day, before the cure was effected. The appetite was much improved during its exhibition.

*Case* 2. This was a man aged 31 years, who, after being troubled with pain in the head for some months, lost the entire power of the lower extremities, their sensibility remaining unimpaired. The power over the sphincters was also lost—the flesh was reduced—the complaint of three month's duration and the cause ascribed to fatigue and cold. Cupping and purging having been premised, the strychnine was exhibited, beginning with the fourth part of a grain three times a day, and gradually increasing the dose to half a grain three times a day, when convulsive twitchings were much complained of, but un accompanied by vertigo or other distressing symptoms. The dose was ultimately augmented to double the above quantity, but severe pain at the scrobiculus cordis was the consequence, with violent spasmodic action of the muscles of the thorax and lower extremities, requiring powerful stimulants. Long and repeated trials were made of the medicine, but no benefit whatever was derived from its administration, and the patient was ultimately discharged uncured. The next case we shall quote in the words of the author.

" *Case* 3. Samuel Ogden, aged 46, admitted 21st June, 1824. He has been paralytic for more than four months, having lost the entire power of the limbs of the right half of the body; and the muscles of that side have not recovered their former bulk and plumpness. He has been addicted to great irregularities in his general mode of living. Complains of pain in front of the head accompanied by vertigo. Speech rather inarticulate. Memory slightly impaired. Urine and fæces are both involuntarily and unconsciously discharged. Appetite indifferent. Pulse feeblc. Countenance somewhat sunk. Six leeches were ordered to each temple, and a blister to the nape of the neck. To take three ounces of the Mistura Sennæ Composita immediately, and to repeat the dose every three hours until the bowels have been freely evacuated. 23d. Head relieved by leeches and blister. Several copious stools procured by a second dose of the purging mixture. To commence with one-twelfth of a grain of strychnia in the form of a pill twice a day. 26th. No effect having been produced, dose increased to one-sixth of a grain. Purging draught to be given on alternate mornings. 29th. Only effect of the medicine is an occasional sensation of heat along the spine. Dose increased to one-fourth of a grain, three times a day. 4th, July. Slight convulsive twitchings of the paralyzed members. More feeling in the bladder and rectum. Half a grain twice a day. 7th July. Slight tetanic symptoms after each dose. Some power over the affected muscles. Half a grain three times a day having produced vertigo, stupor, pain at scrobiculus cordis, irregular convulsive startings both of the sound and affected limbs, tending to syncope, weak pulse, and extreme debility. It was again reduced to twice a day. This dose was continued until the first of August. The patient having improved progressively, until the functions of the bladder and rectum were restored, and he could walk from one end of the large ward to the other, he was discharged.

*Case* 4. This was a spinner, aged 29 years, who, six months previously was seized with loss of power in the lower extremities after bathing whilst the body was much heated with exercise. At the time of admission he was incapable of motion without the aid of crutches, and passed his urine and fæces involuntarily. The spine was free from pain—strength much reduced—appetite bad. The strychnine, in the dose of one-sixth of a grain three times a day, was exhibited, and augmented to a quarter of a grain every four hours, when severe convulsive twitchings took place in the affected limbs. In less than a month he became capable of retaining both his urine and fæces, and of walking from one end of the ward to the other by the aid of a small stick. He was entirely cured.

*Case* 5. Sarah Hilton, aged 36 years, was admitted on the 7th September. This woman had suddenly lost the power of the right side of the body. The attack was preceded by severe pain at the crown of the head, drowsiness, faulty articulation

&c. The intellectual faculties were impaired, as was the power over the sphincters. She was ordered five grains of calomel, followed by a saline aperient, which were repeated, and then the strychnine was exhibited, in the dose of a quarter of a grain every six hours. This was gradually increased till violent twitchings of the paralytic parts ensued, attended by nausea and dyspnœa. These symptoms were relieved by plenty of brandy and water. The medicine was soon afterwards resumed and continued. The power of motion in the paralyzed parts soon began to return—the amendment was rapid—and in about seven weeks the cure was complete.

Passing over a case or two, we shall extract the following without abbreviation.

*Case* 6. Robert Hobson, 38 years of age, finisher, admitted an in-patient, October 6, 1827.

"This is a case of paraplegia, the patient having lost the power over the inferior extremities, rectum, and bladder. He states that he first perceived about four months ago a weakness in his legs, rendering it necessary for him to use considerable effort to drag them along. This debility gradually increased, until at length he became altogether incapable of moving the lower limbs. He has no feeling in them. Head free from pain or giddiness. Pulse regular, appetite impaired. Being desirous of putting the *individual* efficacy of the strychnia to the test, I commenced with the exhibition of the alkali in the proportion of a sixth of a grain twice daily, without previously employing internal or local remedies of any kind. October 10th. No change. Dose to be increased to the fourth of a grain three times in the day. 20th. He considers himself better, having obtained a slight command over the sphincters of the bladder and rectum. On the 14th, he first experienced involuntary twitchings in the inferior extremities, which have been continued at intervals up to the present time. To take half a grain of the alkali twice daily. 28th. He is in excellent spirits, owing to the benefit he has derived from the pills. He can raise the lower limbs to some height from the bed, and also retain at pleasure both his urine and fæces. During the last week, the twitchings have been rather severe, but not painfully so. He is very desirous of persevering with the pills. To continue. November 7th. Since the last report, the patient has been allowed to sit up for several hours during the day. He is surprisingly improved, being able to walk without the aid of a stick from one end of the long ward to the other. Appetite good. Bowels regular. Warmth and sensibility of inferior extremities natural. Half grain pill to be taken three times daily. 26th. The additional half grain excited for some days powerful twitching in the legs and thighs, but in the course of a week or less they became not more severe than was occasioned by half a grain of the alkali taken twice in the day. He is now capable of walking as well as at any former period of his life, and his general health is excellent. It is impossible to describe the gratitude which this patient felt for his restoration to health. He was ordered to be discharged *cured* at the first meeting of the weekly board."

Our author next states the effects of the strychnia in thirteen cases of paralysis ; but, in order to avoid tiresome details of history, he merely mentions the age of the patient, duration of the disease, time of admission, dose of alkali employed, length of time it was continued, and the result. This abbreviation we shall here introduce, as it harmonizes with our own plan of brevity.

" *Case* 11. August 4th, 1825, Elizabeth Taylor, aged 38.

" Has been paralytic on the right side for two months. Attack sudden, accompanied with pain in the head. To commence with the eighth of a grain of strychnia twice daily. Gradually increased to half a grain twice in the day. Continued this dose for three months. Discharged cured.

" *Case* 12. September 2d, 1825, Sarah Whyatt, aged 53.

" Ill six months. Hemiplegia of right side. Fourth of a grain of strychnia to be taken twice daily. Increased to half a grain, at the same intervals. Continued for four months. Discharged relieved.

" *Case* 13. October 4th, 1825, William Johnson, aged 16.

" Paraplegia of six weeks standing. Cause of disease unknown. Sixth of a grain of alkali to be taken twice in the day. Increased to the fourth of a grain. Persevered in its use for two months. Discharged cured.

" *Case* 14. December 30th, 1825, Thomas Griffiths, aged 61.

" About two months ago was seized with hemiplegia of the left side. Former habits intemperate. Head painful. Ten leeches to be applied to temples. Pain in the head removed by leeches. Sixth of a grain of strychnia to be given twice in the day. Increased to half a grain. Continued three months. Discharged relieved.

" *Case* 15. January 4th, 1826, Ann Lloyd, aged 42.

" Has been paralytic on the left side for more than six months. Commenced with the eighth of a grain of alkali. Increased to half a grain three times daily. Continued four months. Discharged cured.

"*Case* 16. January 15th, 1826, Thomas Nott, aged 50.

"Hemiplegia of left side. Experienced the attack about twelve months ago. Sixth of a grain of strychnia to be taken each morning and evening. Increased to a grain and a half during the day. Persevered in the use of the remedy with great regularity for three months. Discharged much relieved.

"*Case* 17. April 2d, 1826, Thomas Ogden, aged 40.

"Has laboured under paralysis of right side for two months. To commence with the sixth of a grain of alkali twice in the day. Increased to half a grain. Continued two months. Discharged cured.

"*Case* 18. September 3d, 1826, John Levers, aged 46.

"Paraplegia of three month's continuance, induced by a severe fall upon the sacrum. Sixth of a grain of strychnia twice daily. Increased to half a grain. Remained in the hospital two months. Discharged cured.

"*Case* 19. August 19th, 1826, Mary Grady, aged 62.

"Lost the power of right side four months ago. Fourth of a grain of alkali twice daily. Increased to half a grain. Three months trial of the medicine. Discharged relieved.

"*Case* 20. October 6th, 1826, William Sicksmith, aged 39.

"Paralytic on right side five months. Fourth of a grain of strychnia twice daily. Increased to a grain and a half in the course of a day. Discharged cured in less than ten weeks from the time of admission.*

"*Case* 21. December 28th, 1826, —— Jones, aged 33.

"Lost power of right arm about year and a half ago. Eighth of a grain of strychnia three times in the day. Gradually increased to half a grain twice daily. Two months trial of alkali. Discharged cured.

"*Case* 22. February 4th, 1827, James Wilson, aged 29.

"Attacked suddenly in March last with paraplegia after exposure to wet and cold. Dose of strychnia to be increased from sixth of a grain to half a grain twice daily. Used this remedy for one month. Discharged cured.

* "This man since experienced an attack of paraplegia, and is now using the strychnia (at his own request) with advantage. March 10th, 1828."

" *Case* 23. November 4th, 1827, James Turner, 42 years of age.

" Has laboured under hemiplegia of left side for five weeks. Commenced with the sixth of a grain twice daily. Dose increased to half a grain. Remained two months in the hospital. Discharged cured.

" The following table will also shew the results of the exhibition of strychnia in *twelve* more cases of paralysis.

| No. | Names. | Age. | Duration of complaint. | Disease. | Result. |
|---|---|---|---|---|---|
| 24 | James Faris, I. P.* | 28 | 3 months. | Hemiplegia of the left side. | Cured. |
| 25 | Charles Walker, I. P. | 44 | 6 months. | Ditto. | Much relieved. |
| 26 | Robert Ashton, H. P. | 54 | 2 years. | Paraplegia. | Slightly relieved. |
| 27 | Margaret Lewis, O. P. | 17 | 2 months. | Ditto. | Cured. |
| 28 | Denis Hagan, H. P. | 44 | 6 weeks. | Hemiplegia of the right side. | Much relieved. |
| 29 | Henry Black, I. P. | 23 | 3 weeks. | Paraplegia from a fall | Cured. |
| 30 | John Allensworth, O. P. | 62 | 12 months. | Hemiplegia of ri't side. | No benefit. |
| 31 | Isabella Gale, I. P. | 33 | 3 months. | Ditto. | Left the house |
| 32 | David Davis, H. P. | 51 | 3 months. | Hemiplegia of the left side. | Much relieved |
| 33 | James Shaw, H. P. | 19 | 4 weeks. | Paraplegia from cold. | Cured. |
| 34 | Owen Morris, O. P. | 54 | 7 months. | Hemiplegia of l'ft side. | Relieved. |
| 35 | Jacob Long, O. P. | 49 | 12 months. | Paraplegia. | Cured. |

### Observations.

Our author thinks and we agree with him, that the foregoing recital is sufficient to prove that strychnia excites a very powerful influence on the system, and that it is entitled to rank as a valuable remedy in the materia medica. It was employed in some cases without benefit, in others with partial advantage—but, in the majority, with complete success. Hence, he conceives, it may justly be considered an *efficacious*, though not a certain remedy in this affection. When cerebral disorganiza-

* " The abbreviations, I. P., H. P., and O. P., denote in-patients, ome-patients, and out-patients."

tion has once taken place, it is, of course, in vain to expect a cure from any medicine. "It is in such cases of paralysis as seem to arise from diminished nervous excitement, that the strychnia is particularly indicated."

"It may be stated here, as a rule of guidance, that whenever hemiplegia supervenes to an apoplectic seizure in persons of a plethoric habit, it is proper to employ bleeding, purging and the ordinary antiphlogistic treatment, before resorting to the use of the strychnia. When the vessels of the head have been freely unloaded, and the quantity and force of the circulating fluid diminished by the above means, there can be little objection to a *cautious* and prudent trial of this remedy. Generally speaking, the strychnia is likely to prove more serviceable in paraplegia, unconnected with spinal disease, than in hemiplegia; though I feel confident, that it will not unfrequently be found an important remedial agent even in hemiplegiac paralysis." 39.

Dr. B. has not tried the strychnine on children, nor would he advise the attempt. The first effects of the medicine in every case, were convulsive twitchings of the paralytic members, and no benefit was derived until this condition of the parts had been produced and continued for some time.—This appears to have been the case with iodine in the hands of that very intelligent and candid physician, Dr. Manson. In Dr. Alderson's experiments, too, with the rhus toxicodendron in paralysis, twitchings and tinglings in the paralytic members appear to have been the first steps towards recovery.—Strychnia does not appear to impair the energy of the stomach, but is rather serviceable in promoting appetite and digestion. Dr. B. recommends the practitioner to commence with not more than the eighth of a grain of strychnine twice in the 24 hours, which quantity is to be gradually increased to a sixth, fourth, or even a half a grain at the same intervals. Any unpleasant symptoms should be the signal for suspending the remedy for a time, when it is to be renewed in slowly augmented doses. "By attention to these points (says our author) although no benefit may accrue from the strychnine, we may be sure that no injury will attend its exhibition." He generally gave it in a pill, with a little conserve of roses.

II. *Strychnia in Chronic Diarrhœa.* Numerous instances of chronic diarrhœa occur among the out-patients of the Manchester Infirmary, some of which obstinately resist every kind of treatment. Under such circumstances, our author was induced to try the strychnia, "and proved a *safe* and *effectual* remedy."

To shew the foundation for this assertion Dr. B. has detailed several cases in illustration. Since he had prepared those pages for the press, he finds that Recamier had also exhibited the alcoholic extract of nux vomica with complete success. Dr. Rummel has also employed strychnia with advantage in chronic blennorrhœa of the rectum.

In his remarks on the cases detailed Dr. B. observes that he does not consider the strychnia a suitable remedy in those instances of diarrhœa which depend upon an evident inflammation of the mucous membrane of the intestines. He particularly recommends the medicine in cases of a *chronic kind*, occurring in persons somewhat advanced in life, and of feeble constitution.

Dr. B. has appended several cases of paralysis treated by brucia an alkali obtained also from the nux vomica, and weaker than the strychnine in the proportion of one to twelve. Ten cases are selected from among others, to show the powers of the brucia. His experience leads him to recommend it as a valuable remedy in paralysis—the action being analogous to that of strychnine, but less powerful—"hence," says he, "it is a preferable remedy in paralytic attacks, accompanied with much cerebral disturbance." It is prudent to commence with the dose of a grain twice a day, which is to be cautiously increased to two grains three or four times a day. Unless a marked advantage accrue from the administration of the remedy in the course of five or six weeks, it ought to be laid aside.—*Medico Chirurgical Rev.*

27. *Cataracts alternating with Diabetes.* (*Winchester county Hospital Reports, in Prov. Med. Gaz. No.* 11.)

The following is a very singular case, and one that would be met with incredulity, were it not guaranteed as it is, by the name of the surgeon and of the hospital.

Eliza Broomfield, æt. 15, remarkably tall and thin, was admitted with cataract in either eye. The pupils were dilated to their utmost, and the crystalline lens were evidently so augmented in bulk as to protrude through the pupillary opening ; its colour was uniformly milky ; the cornea unusually glassy ; the vision so completely lost that the patient could only distinguish most imperfectly light from darkness. Independent of the cataracts, there were dyspnœa, cough, and loss of appetite.

The cataracts had appeared simultaneously fourteen months previous to her admission, and had been completely formed in the surprisingly short period of twenty days. The menses had appeared at the age of eleven, and had flowed with regularity till her thirteenth year, when they disappeared. From having been healthy and robust, she now became very debilitated, increased most astonishingly in stature, and subsequently was attacked with profuse nocturnal perspiration. When twelve months had elapsed an evident amendment took place ; but it proved to be temporary only, and very shortly afterwards the girl began to complain of uneasiness about the head, with vertigo, confusion, and obscurity of vision.

R. Infus. gent, c. ozj. Spt. æth nit. ozss. Tr. camph. c. ozss. ter die sumend. Pil. hyd. gr. ij. omni nocte sumend.

These medicines were continued for upwards of a fortnight with considerable benefit, and on the 20th of February we find that, though the cough was nearly gone, the dyspnœa and appetite were worse. The dilatation of the pupils, bulging, and milky whiteness of each lens were diminished, and the patient could readily detect any substance interposed between her and the light from the window. The medicines were repeated with the addition of an aperient. On the 24th she could discern the flame of a candle at some little distance, but it was now discovered that the urine was too abundant.

" Feb. 28th.—The quantity of urine passsed for the last four days has averaged sixteens pint daily. Its colour is of a greenish yellow, and its taste strongly mellitic. Pulse 65, and feeble ; appetite natural ; dyspnœa not diminished ; the emaciation very considerable ; tongue morbidly clean ; skin dry and scurfy. The cataracts are rapidly disappearing. In a few days the sight was perfectly restored. By a diet of animal food and suitable medicines, the urine was reduced, by the 25th March, to two pints, natural in respect to colour and taste. The sole complaint appeared to be extreme debility, and the patient having recovered her sight returned to her friends. The following account was given by a relative who visited the hospital once a fortnight as an out patient, and was subsequently confirmed by the testimony of the mother of the patient.

" From her narrative, it appears that almost immediately (about four days) after the young woman had returned home, she began to experience a relapse of uneasiness about the head, accompanied with great obscurity of vision, which increased with such rapidity, that before a week had elapsed, she was in a state of utter darkness. To this pitiable condition succeeded her diabetic symptoms, but in a more aggravated form than in her previous attack, together with rapid emaciation and overpowering debility. The quantity of urine evacuated for several days prior to her decease averaged at twenty pints per diem ; but with the accession of these symptoms there was a complete restoration of the natural functions of the eye, which she retained most perfectly to the last moments of her existence. Her death took place four weeks after the return of the diabetes. There had been no post-mortem examination.—*Medico-Chirurgical Review.*

28. *Infanticide.*—We find an interesting memoir on this subject in the *Journ. Gen. de Med.* for November 1829, by J. B. F. HENNEQUIN. It is entitled *Medico-Legal Questions relating to Infanticide.* The interest always attached to this subject induces us to present the article nearly entire to our readers.

A female, R——, accused of infanticide, says, in reply to the questions put to her, at one time, that the child of which she had been delivered had been dead in her womb several days before its expulsion; at another time, that this child died after its birth, in consequence of loss of blood from the umbilical cord, which she was unable to tie, on account of her weakness and fainting away; and that even had she preserved her senses, she would not have tied it, not knowing that such a precaution was necessary.

There being no document at the trial, not even the account of the examination of the body of the infant, performed by the health officer, which furnished suitable data or satisfactory evidence of either of the allegations of the accused, the judge of the second arrondissement of the department of Ardennes put the following questions, to which he wished to have circumstantial answers:

SECOND QUESTION.—*By what signs are we able to ascertain whether a child has come into the world alive? And what are the signs of the death of a child in the womb of its mother?*

ANSWER.—The first part of this question is equally delicate and important: its solution, in order to be positive and free from cavil, ought to rest entirely on an examination of the thorax, lungs, heart, interauricular foramen, the canalis arteriosus, the canalis venosus, the umbilical cord, and the diaphragm.

To live and to breathe are synonymous terms. If the child has not lived, there is no cause for suspicion. Life and respiration being inseparable, whatever in the body of the newly born child shows that respiration has been performed, becomes in itself a proof of life after birth. We may then admit that a child has breathed, and consequently that it came into the world alive, whenever the chest is augmented in size, and changed in form; so that from flat, as it was, it has become arched; when the lungs fill completely the cavity of the chest, and invest almost the entire pericardium; when their colour, of a deep red before breathing, has become clearer, and more or less pale; when the pulmonary cells are filled with air, and give to the organ an emphysematous character; when the lungs, or portions of them, put into a deep vessel, filled with clear and cold water, swim ; when the interauricular foramen is nearly closed ; when the arterial and venous canals are shrunk or obliterated ; when the diaphragm is flattened, &c.

If, after having settled these various points of the respiratory test, we ascertain that the child has reached its full term ; that no diseased condition of the lungs or other viscera exists, susceptible of influencing its vital energies ; that the body presents none or scarcely any of the marks of putrefaction ; that the umbilical cord is shrunk, and there are evidences of the urinary or fœcal excretions having taken place, we are justified in concluding that the child came into the world alive.

As to the signs of the death of the fœtus in the maternal womb, the object of the second part of the second question, they are little more than problematical. The absence of the apparent signs of the child being alive in the womb, does not always prove that it is so, in so positive a manner as to put us beyond the reach of error. In order to arrive at something like certainty on this point, we must class together all the signs of fætal death.

For greater clearness and precision, we must first consider the signs which indicate the death of the child in the still occluded uterus, and then lay down those of a more evident nature, presented by the inspection of the body after birth. In general these signs will be found confirmatory of each other.

The signs, indicative of the death of the child in the uterus, are commemorative, general and particular.

The commemorative signs are all those which apprize us of the causes which could give rise to the death, of which some depend on the mother, and others on the child.

The causes peculiar to the mother are internal or moral, such as great paroxysms of anger, great fright, or chagrin, in fine, all the violent passions ; and external or physical, such as falls, strains, blows on the loins or abdomen, the use of emmenagogue remedies, or of substances to procure abortion ; alarming diseases, such as the phlegmasiæ, convulsions, great hemorrhages.

The causes of the death of the child in the maternal womb, peculiar to itself, are too obscure for us to attempt their enumeration.

The general signs are those which experience has sanctioned as indicative of the death of the fœtus before delivery ; such are the cessation of all movements of the womb, after extraordinary agitation ; an uneasy weight to the mother ; pain in the loins ; the abdomen hanging or falling to the right or left side, according to the attitude taken by the woman ; the uterus in these different movements follows the inclination of the body ; it alone changes its position, without the abdomen also inclining to the recumbent side; when the mother complains of no longer feeling her child move about, the accoucheur by having recourse to the *toucher* at different times, cannot discover any movement, and the uterus descending, compresses the parts which surround it, so that walking becomes difficult for the mother, who believes that the child is dead, and says that it has fallen down; should delivery be retarded, she complains of many distresses, is seized with fever, &c.

These general signs would not constitute a certain prognosis of the death of the child in the uterus, if we did not add to them the particular or special signs by which the proof is made complete.

The particular signs are manifested during labour; they are more or less evident according to the period of the death, whether for a length of time, or immediately before delivery. In the first case, we see the waters of the amnios turbid, holding meconium, and having a fetid odour; a discharge of fetid fluid for a varying length of time; the bones of the cranium moveable, and the cutaneous tissue which covers them, livid, flabby, and easily detached; want of heat and pulsation in the umbilical cord. But we can only judge of this last when the cord is external, or when it forms a turn across the orifice of the uterus.

In the second case, that is, when death has only preceded delivery by a short time, there is no sign of putrefaction of the waters, nor of the skin, but we remark that at the moment in which the head presents at the superior strait, the skin of the cranium does not form the swelling or puffiness which we meet with when it is living; the umbilical arteries are destitute of heat and pulsation, which we ascertain by touching the cord.

All these signs ought to be presented collectively, in order to form a complete proof, otherwise they will not be sufficient to enable us to give a certain prognosis.

The sensible signs presented by the inspection of the body are of a much greater weight. Collectively, they indicate, beyond doubt, the death of the infant in its maternal womb. The pulmonary test practised, as above indicated, might alone suffice to lead us to a definitive judgment. Hence, if we find the thorax narrow, contracted, flattened; the lungs shrunk and dense, and no longer covering the pericardium; if they be of a deep red, and, when put into a deep vessel of pure cool water, sink; if the interauricular foramen, and the canalis arteriosus be free and well open; the umbilical vessels shrunk up; the bladder filled with urine, and the intestines with meconium, we have undoubted proofs that the child has not respired, and, consequently, that it was dead before delivery. The case is still more evident, when the body of the child exhibits marks of putrefaction, exhales a cadaverous odour, allows of the epidermis to be detached, and when the colour of the skin, and especially of that of the abdomen is changed and livid, &c. though the body have been but a short time exposed to the contact of the external air.

It is in the sum of the circumstances just detailed, that the physician must seek for proofs of the death of the fœtus in the maternal womb. To pretend to decide the question after a small number of symptoms, would be evidently exposing one's self to serious mistakes.

Third Question. *Does a neglect to tie the umbilical cord necessarily occasion the death of the new born child by the loss of blood?*

1. The ligature of the umbilical cord is not in every case indispensable. When the circumstances are favourable, it may remain united, without our having to fear any hemorrhage; but we are not, on this account, to conclude that it is always superfluous, since examples are not wanting in which a fatal loss of blood has been the consequences of omitting to perform the operation.

3. Individual circumstances render the ligature of the umbilical cord more or less necessary; hemorrhage is more to be dreaded in a feeble than a robust child; in one who has cried and respired, than in one who is still in a contrary state.

If we cannot positively aver the absolute necessity of the ligature of the umbilical cord, or that the omission of it shall necessarily bring on a fatal hemorrhage, we may say that this precaution is always a good one, and that children, in the case of whom it was neglected, would not be exempt from danger, a fact which very few women are, or ought to be ignorant of, unless they bear a child for the first time.

Fourth Question. *If the death of the child have been caused in consequence of omitting to tie the umbilical cord, by what signs are we to ascertain whether the hemorrhage which ensued was the cause of its death?*

Answer. The authors above cited will also furnish all the materials for this reply. The signs characteristic of the death of a child, after loss of blood, are a pale colour of the whole surface of the body, similar to that presented by wax; paleness of the viscera, discoloration of the muscles; want of blood in the arteries and large veins, and in the auricles and ventricles of the heart, especially in the anterior or right auricle. To these circumstances let us add those which apprize us of the infant's having lived. But we are not to conclude from these signs that there has been umbilical hemorrhage, unless there have existed no other lesion capable of causing a loss of blood, and we see that the body of the child is perfectly well formed, and the umbilical cord neither shrivelled up nor dry, which last would denote that blood had not been supplied to the child in sufficient quantity when it was in the womb of its mother.

We ought to be aware, that even though every thing should prove umbilical hemorrhage, we are not, therefore, to infer an act of criminal premeditation ; the effusion of blood might have been occasioned during the progress of labour, either by too precipitate a detachment of the placenta, or by a laceration of the umbilical cord, especially after a sudden expulsion of the child, and the mother have so fallen into a swoon.—*N. A. Med. and Surg. Journal.*

